# Supplementary material for: Area per player in small-sided games to replicate the external load and estimated physiological match demands in elite soccer players
Source: PLoS One. 2020 Sep 23;15(9):e0229194. doi: 10.1371/journal.pone.0229194 (PMC7510966; doi:10.1371/journal.pone.0229194)
Supplement: S1 Table — The small-sided games with goalkeepers are split for the number of players and pitch size (width x length). The total pitch area and area per player have been calculated. The average number of observations per player for each condition are also reported as mean (max-min). (DOCX) [file pone.0229194.s001.docx]

**S1 Table. Small-sided games with goalkeepers.**

| Number of players | Pitch size | Pitch area | Area per player | Individual sample |
| --- | --- | --- | --- | --- |
|  | **m** | **m^2^** | **m^2^⋅player** | **mean (max-min)** |
| 10*v*10 | 40 x 42 | 1680 | 84 | 8 (14 - 1) |
|  | 55 x 52 | 2860 | 143 | 2 (3 - 1) |
|  | 52.5 x 65 | 3413 | 171 | 2 (4 - 1) |
|  | 65 x 57 | 3705 | 185 | 4 (5 - 1) |
|  | 70 x 65 | 4550 | 228 | 9 (16 - 1) |
|  | 105 x 65 | 6825 | 341 | 2 (3 - 1) |
| 9*v*9 | 40 x 52 | 2080 | 116 | 2 (4 - 1) |
|  | 60 x 40 | 2400 | 133 | 1 |
|  | 52.5 x 65 | 3413 | 190 | 1 (2 - 1) |
| 8*v*8 | 40 x 52 | 1280 | 80 | 2 (2 - 1) |
| 7*v*7 | 35 x 40 | 1400 | 100 | 1 |
|  | 40 x 52 | 2080 | 149 | 4 (8 - 1) |
| 6*v*6 | 40 x 20 | 800 | 67 | 1 |
|  | 40 x 35 | 1400 | 117 | 5 (7 - 2) |
|  | 52 x 40 | 2080 | 173 | 1 |
| 5*v*5 | 40 x 25 | 1000 | 100 | 1 |
|  | 30 x 30 | 900 | 90 | 2 (4 - 1) |
|  | 36 x 40 | 1440 | 144 | 4 (5 - 1) |

The small-sided games with goalkeepers are split for the number of players and pitch size (width x length). The total pitch area and area per player have been calculated. The average number of observations per player for each condition are also reported as mean (max - min).
