# Supplementary material for: Area per player in small-sided games to replicate the external load and estimated physiological match demands in elite soccer players
Source: PLoS One. 2020 Sep 23;15(9):e0229194. doi: 10.1371/journal.pone.0229194 (PMC7510966; doi:10.1371/journal.pone.0229194)
Supplement: S2 Table — The small-sided games without goalkeepers are split for the number of players and pitch size (width x length). The total pitch area and area per player have been calculated. The average number of observations per player for each condition are also reported as mean (max-min). (DOCX) [file pone.0229194.s002.docx]

| Number of players | Pitch size | Pitch area | Area per player | Individual sample |
| --- | --- | --- | --- | --- |
|  | **m** | **m^2^** | **m^2^⋅player** | **mean (max-min)** |
| 10*v*10 | 30 x 40 | 1200 | 60 | 3 (5 - 1) |
|  | 30 x 52 | 1560 | 78 | 6 (8 - 1) |
|  | 40 x 40 | 1600 | 80 | 2 (2 - 1) |
|  | 52.5 x 65 | 3413 | 171 | 2 (2 - 1) |
|  | 95 x 40 | 3800 | 190 | 1 |
|  | 70 x 65 | 4550 | 228 | 4 (5 - 1) |
| 8*v*8 | 35 x 30 | 1050 | 66 | 1 |
|  | 32 x 40 | 1280 | 80 | 2 (2 - 1) |
|  | 35 x 40 | 1400 | 88 | 1 |
|  | 52.5 x 65 | 3413 | 213 | 1 |
| 7*v*7 | 20 x 30 | 600 | 43 | 1 |
|  | 30 x 30 | 900 | 64 | 2 |
|  | 45 x 35 | 1575 | 113 | 4 (7 - 1) |
|  | 33 x 52 | 1716 | 123 | 1 |
|  | 35 x 55 | 1925 | 138 | 1 |
|  | 65 x 52.5 | 3413 | 244 | 2 (2 - 1) |
| 6*v*6 | 35 x 30 | 1050 | 87.5 | 4 (7 - 1) |
|  | 40 x 30 | 1200 | 100 | 1 |
|  | 45 x 30 | 1350 | 112 | 1 (2 - 1) |
|  | 60 x 30 | 1800 | 150 | 1 |
|  | 65 x 52.5 | 3412 | 284 | 1 |
| 5*v*5 | 30 x 30 | 900 | 90 | 1 (2 - 1) |
|  | 35 x 30 | 1050 | 105 | 4 (6 - 1) |
|  | 40 x 25 | 1000 | 100 | 2 (3 - 1) |
|  | 45 x 25 | 1125 | 112.5 | 1 |
|  | 45 x 30 | 1350 | 135 | 1 |
|  | 65 x 52.5 | 3412 | 341 | 2 (3 - 1) |
| 4*v*4 | 30 x 20 | 600 | 75 | 1 |
|  | 30 x 30 | 900 | 112 | 2 (4 - 1) |
|  | 40 x 35 | 1400 | 175 | 3 (4 - 1) |
| 3*v*3 | 20 x 20 | 400 | 67 | 1 |
|  | 30 x 20 | 600 | 100 | 1 |
|  | 30 x 25 | 750 | 125 | 1 |

**S2 Table. Small-sided games without goalkeepers.**

The small-sided games without goalkeepers are split for the number of players and pitch size (width x length). The total pitch area and area per player have been calculated. The average number of observations per player for each condition are also reported as mean (max - min).
