# Supplementary material for: Area per player in small-sided games to replicate the external load and estimated physiological match demands in elite soccer players
Source: PLoS One. 2020 Sep 23;15(9):e0229194. doi: 10.1371/journal.pone.0229194 (PMC7510966; doi:10.1371/journal.pone.0229194)
Supplement: Attachment — Submitted filename: response to reviewers.docx [file pone.0229194.s005.docx]

PONE-D-20-02888R1
Area per player in small-sided games to replicate the external load and estimated physiological match demands in elite soccer players
PLOS ONE

Additional Editor Comments (if provided):

Please, one further effort to address remaining Reviewer 3's issues.

Reviewer #3: General Comments
I commend the Authors for their thorough and well-considered revision. I have only minor corrections, but believe that this manuscript is suitable for publication.

**We would to thank the Reviewer for his useful comments that help us to improve the manuscript.**

Specific Comments
Introduction
Page 3 Line 70 – change “controversial” to “conflicting”.

**As suggested, we changed “controversial” to “conflicting”. Please see text.**

Page 4 Line 75 – overall, I commend the Authors for providing a more balanced (and evidence-based) account of Metabolic Power. However, in saying that the model is “…used to estimate the energy cost of high-intensity running activities…”, this implies that it is only useful for this purpose, whereas in fact it applies across all intensities. So, please change “the energy cost of high-intensity running activities in team sports” to “the energy cost of locomotion in team sports”.

**We thank the Reviewer for his comment. We strongly agree with him and we reworded the sentence. Please see text.**

Reviewer #4: All my comments have been satisfactorily adressed. In my opinio, the manuscript can be opublished in its current form.

**We would to thank the reviewer for his/her comment and for his/her previous suggestions that help us to improve the manuscript.**
